# Application of cryopreservation to tooth germ transplantation for root development and tooth eruption

**DOI:** 10.1038/s41598-021-88975-1

**Published:** 2021-05-04

**Authors:** Xinghan Li, Megumi Nakamura, Weidong Tian, Yasuyuki Sasano

**Affiliations:** 1 Division of Craniofacial Development and Tissue Biology, Tohoku University Graduate School of Dentistry, 4-1 Seiryo-machi, Aoba-ku, Sendai, 980-8575 Japan; 2 State Key Laboratory of Oral Diseases, West China Hospital of Stomatology, Sichuan University, Sichuan, China; 3 National Engineering Laboratory for Oral Regenerative Medicine, West China Hospital of Stomatology, Sichuan University, Sichuan, China; 4 Department of Oral and Maxillofacial Surgery, West China Hospital of Stomatology, Sichuan University, Sichuan, China; 5 Department of Stomatology, Shenzhen University General Hospital, Shenzhen, China

**Keywords:** Oral surgery, Organ transplantation

## Abstract

We cryopreserved mouse tooth germs with widely open cervical margins of the enamel organ to overcome difficulties in cryoprotectant permeation and tested their efficacy by transplanting them into recipient mice. The upper right first molar germs of 8-day-old donor mice were extracted and categorized into the following four groups according to cryopreservation time: no cryopreservation, 1 week, 1 month, and 3 months. The donor tooth germs were transplanted into the upper right first molar germ sockets of the 8-day-old recipient mice. The upper left first molars of the recipient mice were used as controls. The outcome of the transplantation was assessed at 1, 2, and 3 weeks after transplantation. Stereomicroscopic evaluation revealed that most of the transplanted teeth erupted by 3 weeks after transplantation. Micro-computed tomography analysis revealed root elongation in the transplanted groups as well as in the controls. There was no significant difference between the cryopreserved and non-cryopreserved transplanted teeth, but the roots of the cryopreserved teeth were significantly shorter than those of the control teeth. Histological examination revealed root and periodontal ligament formations in all the transplanted groups. These results suggest that the transplantation of cryopreserved tooth germs facilitates subsequent root elongation and tooth eruption.

## Introduction

Autotransplantation is a treatment option for missing teeth and is a clinically effective strategy that has been reported to result in successful outcomes^1,2^. Unlike other treatment approaches such as dental implants and bridges, autotransplanted teeth are naturally biocompatible, and the procedure does not damage adjacent teeth. However, its usefulness is limited because a healthy donor tooth is not always available when required.

Cryopreservation enables donor teeth to be stored in a viable condition for extended periods, and the application of this technique to tooth transplantation has considerable potential for further expansion. Successful cryopreservation of cells and tissues requires the prevention of ice crystal formation during the freezing process as this can damage cellular structures. For this purpose, cryoprotective agents such as dimethylsulfoxide (DMSO) are frequently used. However, in the cryopreservation of mature teeth, difficulty has been reportedly in maintaining the biological viability of the pulp tissue owing to the cryoprotective agents barely penetrating the pulp cavity through the narrow apical foramen, which is surrounded by hard tissue^3–5^. This finding is consistent with that of studies that showed that autotransplantation of cryopreserved immature teeth achieved higher success rates than that of mature teeth. Several studies in rats have demonstrated that subcutaneously transplanted, cryopreserved immature teeth are comparable with freshly isolated, non-preserved teeth with respect to periodontal and pulp tissue regeneration^6–9^. In addition, no differences in pulpal regeneration and revascularization of the pulp cavity have been reported between cryopreserved mature apicoectomized and immature teeth after transplantation in dogs^10^.

Collectively, these observations led us to investigate the possibility of transplanting tooth germs cryopreserved prior to root formation. At this stage of tooth germ development, the cervical margin of the enamel organ is wide open, which facilitates much better penetration of cryoprotective agents; thus, we hypothesized that root formation and tooth eruption could be achieved after transplantation of cryopreserved tooth germs. The present study was aimed at testing this hypothesis. Cryopreserved tooth germ transplantation outcomes were quantitatively monitored using micro-computed tomography (CT) and qualitatively assessed using histological analysis in mice.

## Methods

### Experimental animals

All the experimental procedures conformed to the Regulations for Animal Experiments and Related Activities at Tohoku University and were reviewed and approved by the Institutional Laboratory Animal Care and Use Committee of Tohoku University (approval No. 2015DnA-059-02). The animal studies conducted in this research were performed in compliance with the ARRIVE guidelines. Eight-day-old C57BL/6 mice were obtained from Japan SLC, Inc. (Hamamatsu, Japan), and used as both donors and recipients.

### Experimental design

A total of 120 tooth germ specimens were categorized into the following five groups (with 24 specimens in each group): Control (under physiological conditions), Fresh (non-cryopreserved transplants), Cryo1w (1-week cryopreserved transplants), Cryo1m (1-month cryopreserved transplants), and Cryo3m (3-month cryopreserved transplants). These were sampled at 1, 2, and 3 weeks after transplantation (W1, W2, and W3, respectively), and eight tooth germs (or teeth) were examined at each time point.

### Cryopreservation of donor tooth germs

The donor mice were euthanized with an overdose of isoflurane (Fujifilm Wako Pure Chemical Corporation, Osaka, Japan) by inhalation, and their upper right first molar germs were carefully extracted. The extracted donor tooth germs were divided into the following four groups according to cryopreservation storage time: Fresh (no cryopreservation), Cryo1w (1 week), Cryo1m (1 month), and Cryo3m (3 months). Donor tooth germs were immersed in a BGjb (Thermo Fisher Scientific, Waltham, Massachusetts, USA) medium containing 10% DMSO in cryogenic vials for 10 min at room temperature. The vials were placed in a freezing container and frozen at a rate of 1 °C/min from room temperature to − 80 °C and were subsequently stored in liquid nitrogen at − 196 °C until use.

### Transplantation procedure

Cryopreserved tooth germs were rapidly thawed in a 37 °C water bath and rinsed with physiological saline. Recipient mice were intraperitoneally anesthetized using a mixture of medetomidine chloride (0.3 mg/kg), midazolam (4 mg/kg), and butorphanol tartrate (5 mg/kg), and their upper right first molar germs were extracted. The thawed donor tooth germs were transplanted into the recipient sockets. The donor tooth germs in the Fresh group were immediately transplanted after extraction without cryopreservation. The upper left first molar germs of the Fresh group of recipient mice were used as controls.

### Stereomicroscopic evaluation

Recipient mice were euthanized using an overdose of isoflurane by inhalation at W1, W2, or W3 after transplantation. Maxillae, including the transplanted and control teeth, were resected and fixed in 4% paraformaldehyde in 0.1 M phosphate buffer. The eruption of the transplanted teeth was evaluated under a stereomicroscope, and the erupted teeth were enumerated. The erupted teeth were defined as those that erupted to a level higher than half the crown height of the adjacent second molar.

### Micro-CT analysis

Micro-CT images of the paraformaldehyde-fixed maxillae containing the transplanted and control teeth were obtained under standardized settings (80 kV, 120 μA, 8 μm/pixel) using a Scan Xmate-E090 (Comscantecno Co., Ltd., Yokohama, Japan). The Fiji software was used to generate three-dimensional images^11^. All slices containing transplanted and control teeth were extracted from the volume images, and regions other than the teeth were removed. Visualization was performed using the 3DViewer plugin. The root lengths of the transplanted and control teeth were three-dimensionally measured using Fiji^11^. The cemento-enamel junction plane resliced at three points on the junction (Fig. 2d) was set as a reference plane, and the distance between the reference and parallel planes passing through the farthest point of the longest root was calculated (Fig. 2e) as previously described^12^. CT images were also used to determine the number of roots in the control and transplanted teeth.

### Histology

After micro-CT scanning, maxillae were decalcified for 3 weeks in 10% EDTA in 0.01 M phosphate buffer and subsequently dehydrated through a graded ethanol series, embedded in paraffin, and cut into 5-μm thick sections. The sections were processed for hematoxylin–eosin or tartrate-resistant acid phosphatase (TRAP) staining. For detecting TRAP activity, the sections were incubated in a mixture of 0.4 mM naphthol AS-BI phosphate (Nacalai Tesque, Inc., Kyoto, Japan) and 75 mM L( +)-tartaric acid (Fujifilm Wako Pure Chemical Corporation) in 0.1 M sodium acetate buffer (pH 5.0) for 30 min at 37 °C. Next, the sections were immersed in the same buffer containing 0.1% pararosaniline chloride (Fujifilm Wako Pure Chemical Corporation) until a red color developed, rinsed with distilled water to stop the reaction, and counterstained with 1% methyl green.

### Statistical analysis

Statistical analysis was performed using SPSS version 22.0 (IBM Corp., Armonk, NY, USA). Root lengths were compared using the Kruskal–Wallis test, followed by the Mann–Whitney U test. The significance level was adjusted to *P* < 0.05/10 = 0.005 with Bonferroni correction.

## Results

### Tooth eruption levels

The eruption of transplanted teeth was evaluated under a stereomicroscope. More than 50% of the transplanted tooth germs in any group ultimately erupted (Table 1), but neither the controls nor the transplanted teeth erupted at W1. All the controls and nearly all the transplants in the Fresh group erupted by W2, but the cryopreserved transplants exhibited delayed tooth eruption. The number of erupted teeth tended to decrease as the cryopreservation period increased. At W3, all the controls and transplants in the Fresh group had erupted. The number of erupted teeth at W3 increased from that at W2 in the cryopreservation groups, except for the teeth in the Cryo1w group.

**Table 1:** Numbers of erupted teeth after transplantation (n = 8/group for each week).

Group	W1	W2	W3
Control	0	8	8
Fresh	0	7	8
Cryo1w	0	6	4
Cryo1m	0	4	7
Cryo3m	0	1	5

### Tooth morphology and periodontal tissue formation

**Figure 1:**
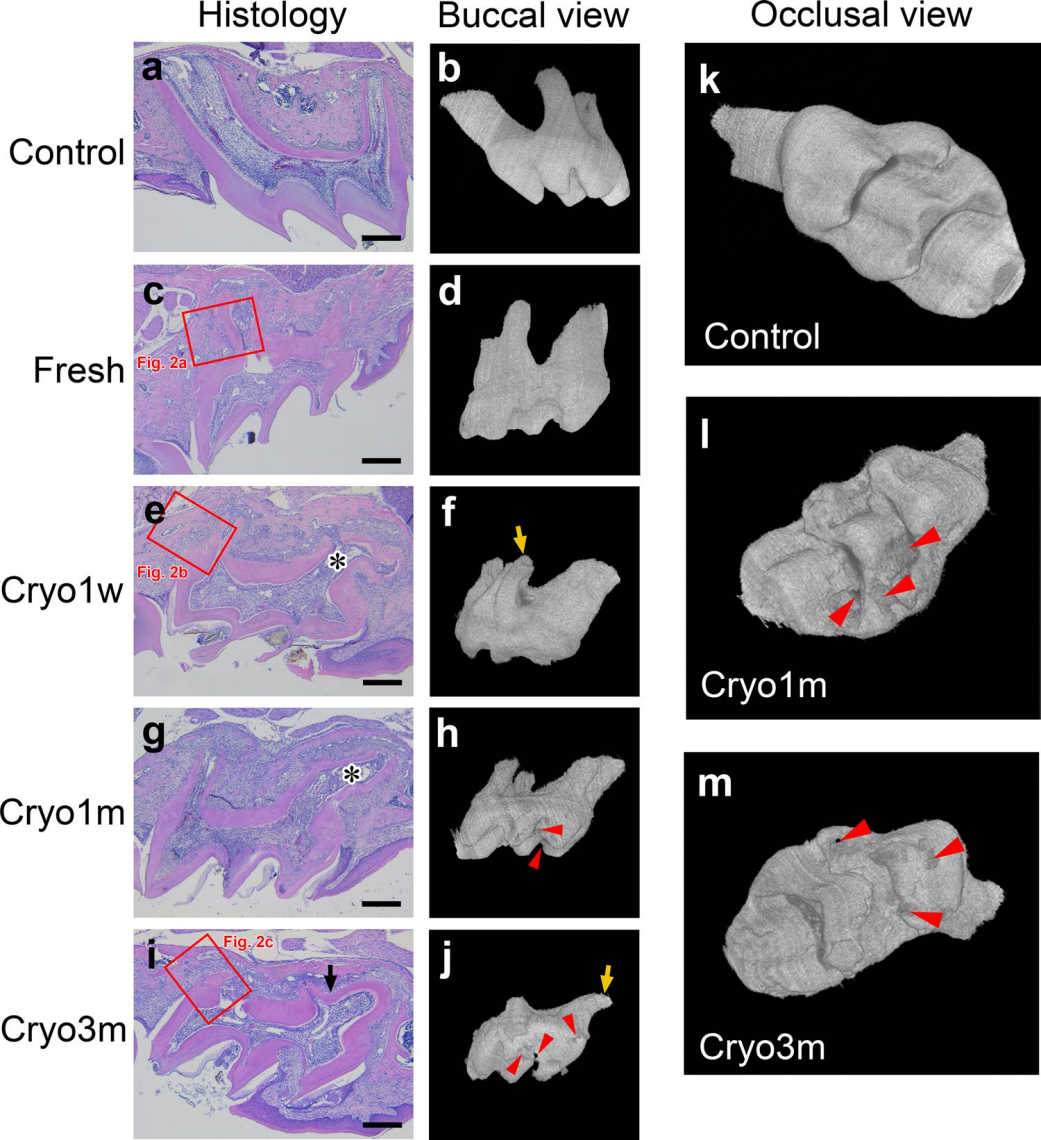
Histologies of the transplanted and control teeth at W3 in the (**a**) Control, (**c**) Fresh, (**e**) Cryo1w, (**g**) Cryo1m, and (**i**) Cryo3m groups. The arrow indicates an abnormally furcated root; and the asterisk, an abnormally bent root. Hematoxylin–eosin staining, scale bar = 300 μm. Three-dimensional micro-computed tomography images of the buccal and occlusal views of the transplanted and control teeth at W3 in the (**b**,**k**) Control, (**d**) Fresh, (**f**) Cryo1w, (**h**,**l**) Cryo1m, and (**j**,**m**) Cryo3m groups are shown. Three-dimensional reconstruction from micro-CT slices and visualization were performed using the Fiji software with the 3DViewer plugin (ImageJ versions 1.53c, https://imagej.net/Fiji). The yellow arrow indicates an abnormally bent root; and the red arrowhead, a dent in the crown.

The tooth germs from the 8-day-old mice that we used for transplantation contained no roots that had completed the crown contour formation. Thus, the three-dimensional micro-CT and histological analyses revealed that regardless of whether the transplant was cryopreserved or not, root elongation occurred in all the transplanted groups as well as in the controls (Fig. 1). However, the crown surfaces of the three-dimensionally constructed cryopreserved transplants were rough, and some of them had dents in the crown (Fig. 1h,j,l,m). The cryopreserved transplanted teeth tended to have an insufficient number of roots (Fig. 1j) and to bend the roots (Fig. 1f,j). Histological examination also revealed that the root morphologies of the transplants were deformed and the walls of their pulp chambers and canals were rugged (Fig. 1c,e,g,i) compared with those of the controls (Fig. 1d). Most of the transplanted tooth roots were abnormally bent (Fig. 1e,g) and furcated (Fig. 1i).

**Figure 2:**
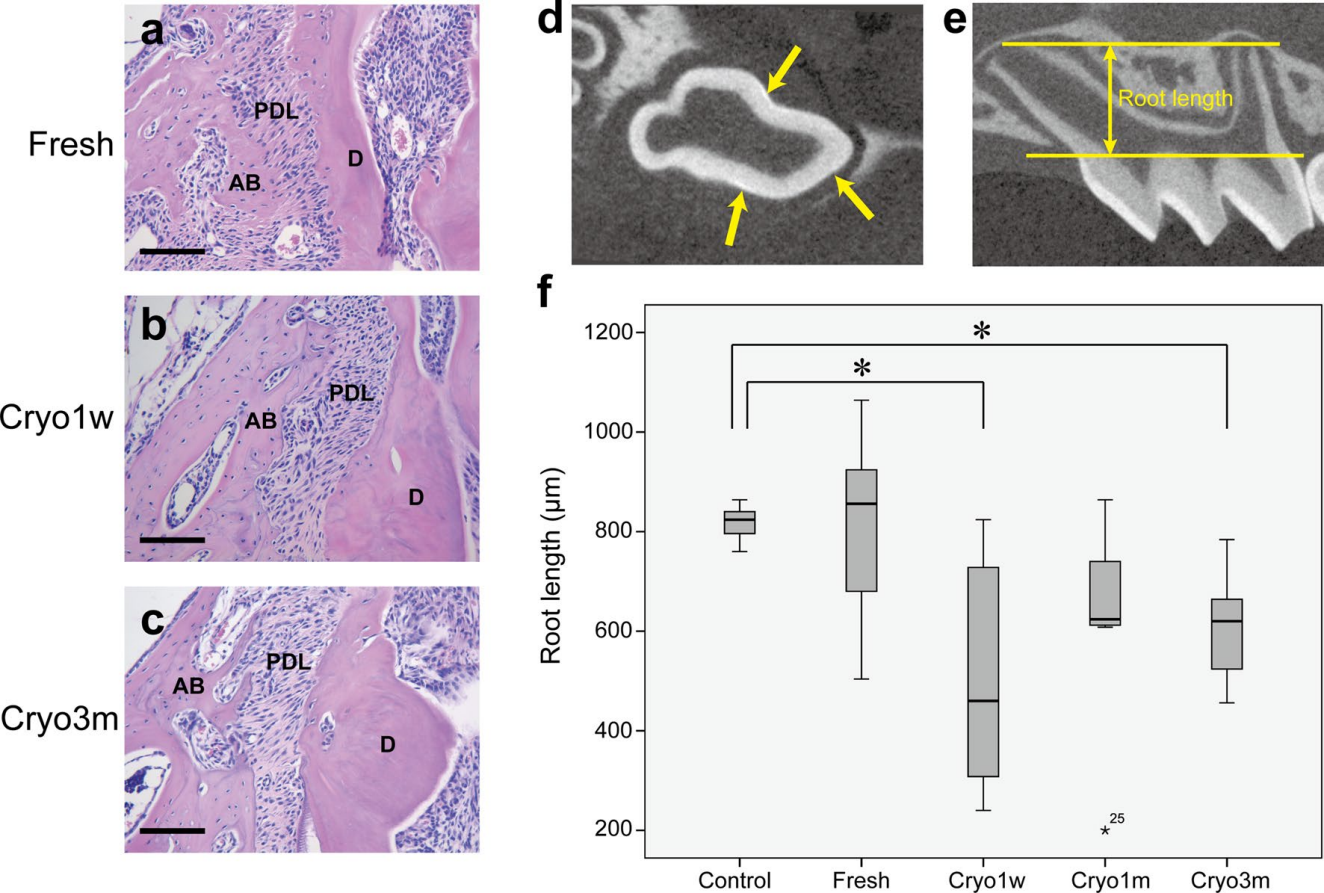
(**a**–**c**) Enlargement of the boxed areas in Fig. 1. Histologies of the periodontal ligaments in the (**a**) Fresh, (**b**) Cryo1w, and (**c**) Cryo3m groups. *PDL* periodontal ligaments; *AB* alveolar bone; *D* dentin. Hematoxylin–eosin staining, scale bar = 100 μm. (**d**) A micro-CT image of a control tooth. The cemento-enamel junction plane resliced at three points on the junction was set as a reference plane. (**e**) A micro-CT image of a control tooth at W3. The distance between the reference and parallel planes passing through the farthest point of the longest root was measured as the root length. (**f**) Comparison of the longest root lengths between the transplanted and control groups. *P < 0.005 using a Kruskal–Wallis test followed by the Mann–Whitney *U* test with Bonferroni correction.

Periodontal ligament (PDL) formation between the cementum and alveolar bone was identified in all the transplanted groups. The PDL cells were regularly arranged in both the transplanted and control groups (Fig. 2a–c).

### Length and number of roots

The length of the longest root of each tooth was quantitatively evaluated using micro-CT analysis, and the data from eight samples at W3 were statistically analyzed for each group, namely the Fresh, Cryo1w, Cryo1m, Cryo3m, and Control groups (Table 2 and Fig. 2f). The roots of the cryopreserved transplants in the Cryo1w and Cryo3m groups were significantly shorter than those of the Control group (*P* = 0.001 for both). No significant differences were observed between the control teeth and transplants of the Fresh and Cryo1m groups (Fresh, *P* = 0.423; Cryo1m, *P* = 0.024), between the fresh and cryopreserved transplants (Cryo1w, *P* = 0.014; Cryo1m, *P* = 0.153; and Cryo3m, *P* = 0.035), and between the transplants that were cryopreserved for 1 week, 1 month, and 3 months (Cryo1w and Cryo1m, *P* = 0.395; Cryo1w and Cryo3m, *P* = 0.442; and Cryo1m and Cryo3m, *P* = 0.596). The number of roots was also assessed from the micro-CT images. The control teeth had three roots, and one or two roots were missing in some transplanted teeth, both with and without cryopreservation (Table 3).

**Table 2:** Longest root lengths (μm) at W3 (n = 8).

Group	Subject number								Mean	SD
	1	2	3	4	5	6	7	8		
Control	864	768	760	848	824	832	824	824	818	36.2
Fresh	544	504	904	1064	816	856	856	944	811	192.7
Cryo1w	712	744	824	240	560	288	360	328	507	231.3
Cryo1m	200	608	632	864	656	616	824	616	627	199.6
Cryo3m	632	672	576	784	472	656	456	608	607	107.3

**Table 3:** Numbers of transplanted teeth at W3 classified according to their root number (n = 8 for each group).

Group	Three roots	Two roots	One root
Control	8	0	0
Fresh	7	1	0
Cryo1w	7	1	0
Cryo1m	5	2	1
Cryo3m	5	3	0

### Degenerative tissue in the pulpo-dentinal complex

**Figure 3:**
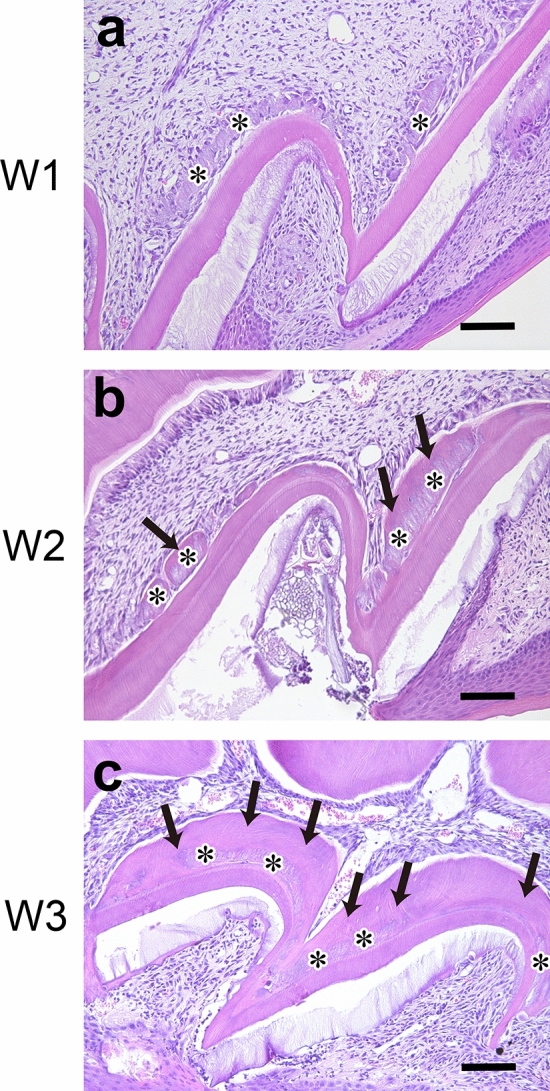
Histology of transplanted tooth crowns at (**a**) W1, (**b**) W2, and (**c**) W3. The asterisk indicates degenerative tissues; and arrow, the dentin layer on the pulp side of the degenerative tissue. Hematoxylin–eosin staining, scale bar = 50 μm.

Pulp vascularization was identified in all the transplanted teeth, and odontoblasts were arranged on the dentin surfaces of their root canals. However, degenerative tissue was observed on the dentin surface of the pulp chamber corresponding to the site of the odontoblasts in all the transplanted groups, with and without cryopreservation at W1 (Fig. 3a). The degenerative tissue was surrounded by dentin in the transplanted teeth at W2 (Fig. 3b), and the dentin layer on the pulp side of the degenerative tissue at W3 was thicker than that at W2 (Fig. 3c).

### Enamel development and multinucleated cells

**Figure 4:**
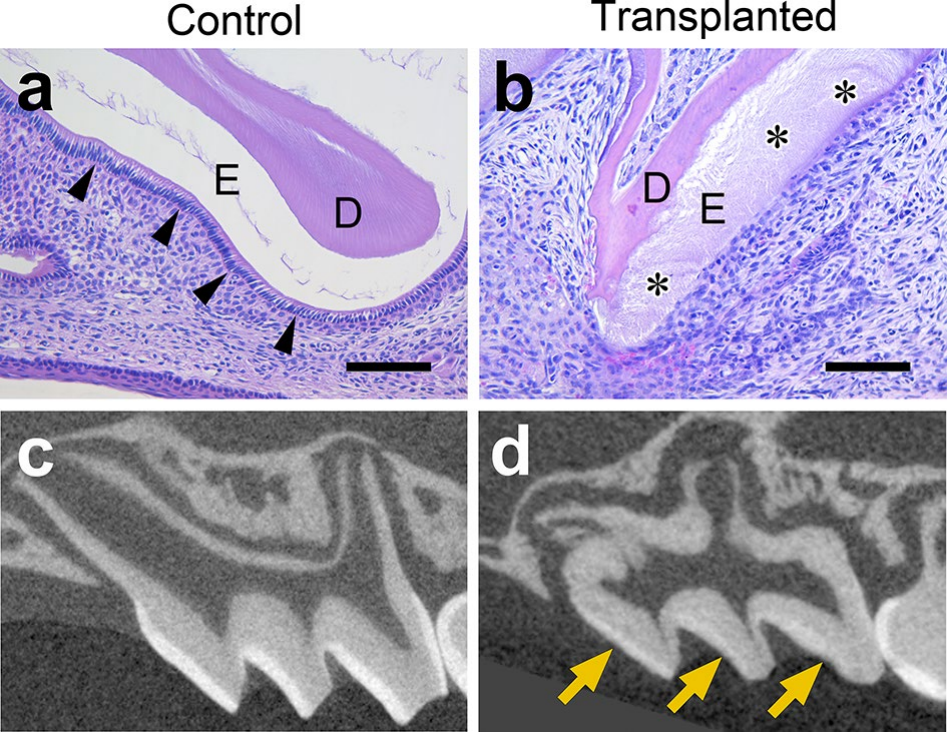
(**a**) A crown cusp of a control tooth at W1. The enamel matrix is barely present, and ameloblasts are in their maturation stage. (**b**) The crown cusp of a cryopreserved, transplanted tooth at W1. The enamel matrix remains, and ameloblasts are absent. *E* enamel, *D* dentin. The arrowheads indicate the ameloblasts; and the asterisk, the enamel matrix. Hematoxylin–eosin staining, scale bar = 100 μm. Micro-CT images of control (**c**) and transplanted teeth (**d**) at W3. The high-density enamel layer is thick in (**c**) but thin in (**d**). Yellow arrows indicate the thin enamel layer.

Histological examination revealed that the calcifying enamel matrix was barely observed during development in the control teeth (Fig. 4a), while it partially remained in all the transplanted groups, even after decalcification (Fig. 4b). Ameloblasts were not found on the enamel surface of both the cryopreserved and non-cryopreserved transplants at W1 (Fig. 4b), while ameloblasts in their maturation stage were observed in the controls (Fig. 4a). In addition, micro-CT images showed a thin, high-density layer on the crown surfaces of the transplanted teeth but a thicker layer in the controls (Fig. 4c,d).

**Figure 5:**
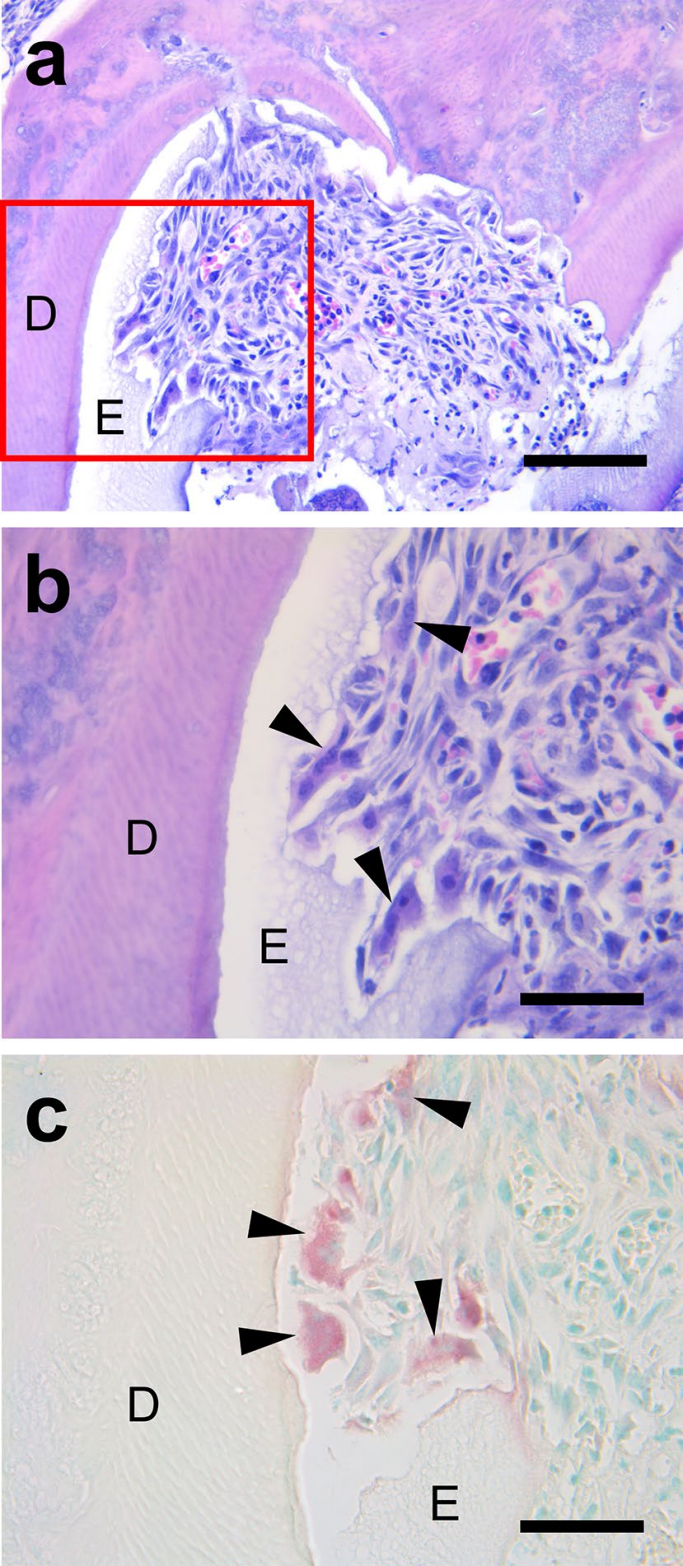
(**a**) Histology of a transplanted tooth crown at W3 in the Cryo1w group. The enamel matrix remains. *D* dentin; *E* enamel. Hematoxylin–eosin staining, scale bar = 100 μm. (**b**) Enlargement of the boxed area in (**a**). Several multinucleated cells are located in the lacunae on the crown surface. *D* dentin, *E* enamel. The arrowheads indicate multinucleated cells. Hematoxylin–eosin staining, scale bar = 50 μm. (**c**) The same area in (**b**) in a consecutive section. TRAP**-**positive multinucleated cells are identified. *D* dentin, *E*, enamel. The arrowheads indicate TRAP-positive multinucleated cells. TRAP staining, scale bar = 50 μm.

Some transplanted teeth with and without cryopreservation exhibited a rough crown surface where multinucleated cells and their TRAP activities were identified in resorption lacunae (Fig. 5). TRAP**-**positive multinucleated cells were not found in the control teeth.

## Discussion

The findings of the present study demonstrated that transplanted cryopreserved tooth germs develop roots and erupt. Studies have examined the possibility of cryopreservation of dental pulp cells^13–15^, PDL cells^16–20^, pulp tissue^5,19,21,22^, and even the whole teeth^2,6–9,23^. On the basis of these reports, cryopreservation appears to have little negative influence on PDL cells, whose viability and function were maintained after cryopreservation and contrasted with the poor viability and lower survival rates of pulp cells. These results imply that permeation of cryoprotective agents into the pulp cavity is a key factor in successful cryopreservation. In this study, we cryopreserved tooth germs to provide preliminary evidence for the feasibility of cryopreservation, and our results indicate that such transplanted, cryopreserved tooth germs can erupt and maintain their functionality. This, in turn, suggests that cryoprotective agents had permeated into the dental papilla, resulting in high cell viability and successful development of transplanted tooth germs.

We investigated the eruption frequency, root length, number, and morphology of the transplanted teeth. Compared with the Fresh group, the cryopreserved teeth exhibited delayed tooth eruption and decreased number of roots with increased cryopreservation time. However, while there was no statistically significant difference in root length between the Fresh and cryopreserved groups, root deformities were identified in both the fresh and cryopreserved teeth. These findings suggest that cryopreservation leads to delayed tooth eruption and reduced number of roots but does not affect root length; however, the deformation may be caused by transplantation and not cryopreservation.

The eruption rate of the teeth decreased from W2 to W3 only in the Cryo1w group. This may have been caused by a technical issue during tooth transplantation. Histological examination and micro-CT revealed that most of the teeth in the Cryo1w group at W3 were distally inclined, while the teeth in the other groups were transplanted in the proper orientation. Tooth germ transplantation requires a sophisticated surgical technique because a tooth germ with no root is easily rotated within a tooth socket during surgery. The success of the transplantation relies on the insertion of the donor tooth germ in the appropriate orientation within a recipient socket.

Odontoblasts were present beneath the pre-dentin surface in the pulp cavity under physiological conditions. In all the transplanted teeth at W1, regardless of cryopreservation, degenerative tissues were found in the putative region of the odontoblast layer of the crown, although odontoblasts in the root were observed to be well organized. At W2 and W3, newly formed dentin was found between the pulp and the degenerative tissue. On the basis of the histological examination findings, the original odontoblasts may have degenerated as a result of severe environmental stresses from events such as extraction and transplantation, and new odontoblasts may have subsequently differentiated from dental pulp stem cells within 2 weeks after transplantation and may have secreted a dentin matrix on the pulp side of the degenerative tissue.

Micro-CT analysis findings showed the presence of a thin enamel layer on the transplanted teeth, which suggests a possible problem with enamel calcification. In addition, a residual enamel matrix was found in the histological sections of the decalcified transplanted teeth, which suggests a delay in the progress of enamel calcification. Ameloblasts were not found on the surface of the crown, and the loss of ameloblasts may have led to failure in the maturation of the enamel matrix after transplantation. TRAP-positive multinucleated odontoclast-like cells were prominent in the crowns of the transplanted teeth at W2 and W3, and some were observed in the enamel matrix. These odontoclast-like cells may have resorbed the immature enamel matrix.

We have demonstrated that cryopreserved tooth germs can develop both roots and periodontal tissues and then erupt, which indicates that the cryopreservation of tooth germs may be a promising strategy for clinical application. The results presented in this study suggest that transplantation of bioengineered tooth germs could result in the functional recovery of lost teeth in the not-too-distant future.
